# Viability and Contractility of Rat Brain Pericytes in Conditions That Mimic Stroke; an *in vitro* Study

**DOI:** 10.3389/fnins.2019.01306

**Published:** 2019-12-05

**Authors:** Mohammed Heyba, Lulwa Al-Abdullah, Andreas W. Henkel, Zeinab Sayed, Slava A. Malatiali, Zoran B. Redzic

**Affiliations:** Department of Physiology, Faculty of Medicine, Kuwait University, Kuwait City, Kuwait

**Keywords:** pericytes, stroke, no-reflow, cytokines, viability, contractility, oxygen glucose deprivation

## Abstract

Reopening of the cerebral artery after occlusion often results in “no-reflow” that has been attributed to the death and contraction (*rigor mortis*) of pericytes. Since this hypothesis still needs to be confirmed, we explored the effects of oxygen glucose deprivation (OGD) on viability and cell death of primary rat pericytes, in the presence or absence of neurovascular unit-derived cytokines. Two morphodynamic parameters, single cell membrane mobility (SCMM) and fractal dimension (D_f_), were used to analyze the cell contractions and membrane complexity before and after OGD. We found a marginal reduction in cell viability after 2–6 h OGD; 24 h OGD caused a large reduction in viability and a large increase in the number of apoptotic and dead cells. Application of erythropoietin (EPO), or a combination of EPO and endothelial growth factor (VEGF_A1−165_) during OGD significantly reduced cell viability; application of Angiopoietin 1 (Ang1) during OGD caused a marginal, insignificant increase in cell viability. Simultaneous application of EPO, VEGF_A1−165_, and Ang1 significantly increased cell viability during 24 h OGD. Twenty minutes and one hour OGD both significantly reduced SCMM compared to pre-OGD values, while no significant difference was seen in SCMM before and after 3 h OGD. There was a significant decrease in membrane complexity (D_f_) at 20 min during the OGD that disappeared thereafter. In conclusion, OGD transiently affected cell mobility and shape, which was followed by apoptosis in cultured pericytes. Ang1 may have a potentiality for preventing from the OGD-induced apoptosis. Further studies could clarify the relationship between cell contraction and apoptosis during OGD.

## Introduction

Pericytes wrap capillaries and express contractile proteins, so they could potentially regulate capillary diameter by constricting or relaxing their processes (Winkler et al., 2017). Whether or not they really control cerebral blood flow (CBF) *in vivo* is not clear. Local CBF is controlled by changes in smooth muscle tone around cerebral arterioles (Attwell et al., 2010). A concept has emerged from an *in vivo*/*in situ* study by Hall et al. (2014), which demonstrated that an increase in neuronal activity dilated neighboring capillaries before dilating arterioles and was accompanied by morphological changes in pericytes' shapes that were consistent with relaxation. A study that used loss-of-function pericyte-deficient mice has shown that pericyte degeneration reduces capillary blood flow responses to neuronal activity (Kisler et al., 2017). These findings have been challenged by studies, which revealed that brain pericytes were not able to contract since the contractile proteins, such as alpha smooth muscle actin (aSMA), was only present in vascular smooth muscle cells (Hartmann et al., 2015; Hill et al., 2015; Wei et al., 2016).

Conflicting evidence exists regarding the role of pericytes in “no-reflow” following reperfusion of the occluded artery. Peppiatt et al. (2006) found that brain pericytes in mice remained contracted after the middle cerebral artery (MCA) was reopened, following 2 h occlusion. Hall et al. (2014) reported that brain pericytes die rapidly following cerebral ischemia and their processes subsequently contract (*rigor mortis*), which causes constriction of the capillaries.

To clarify whether or not conditions that occur during cerebral ischemia *in vivo* cause rapid death and/or a long contraction of brain pericytes, we cultured primary rat pericytes and then measured their viability and motility at different time points during oxygen glucose deprivation (OGD).

## Materials and Methods

### Animals

Primary cultures of pericytes were obtained from 1 to 2-month-old male and female Sprague Dawley rats weighting 200–220 g. Animals were supplied by Animal Resource Center (ARC), Health Science Center (HSC), Kuwait University.

The study was carried out in accordance with the guidelines of laboratory animal care in HSC, Kuwait University, which are based on principles of the Office of Laboratory Animal Welfare, National Institutes of Health^1^, and on Animal Research: Reporting of *in vivo* Experiments recommendations^2^.

### Primary Culture of Rat Brain Pericytes

Primary pericyte cultures were generated according to a protocol for rat brain endothelial cells (BECs) isolation (Abbott et al., 2012). For details on how this protocol was amended to produce pericytes, see Supplemental Figure 1. Experiments that tested cell viability were performed on >90% confluent cultures, while experiments that analyzed contractility of pericytes were performed on cultures at <50% confluence.

### Immunocytochemistry

Unless stated otherwise all antibodies were from Abcam, UK. Following fixation, permeabilization and blocking, cells were incubated overnight at 4°C with the following six primary antibodies (1:100 dilution) in PBS/0.1% Tween20/1% FCS: (1) Rabbit polyclonal antibody raised against purified rat NG2 chondroitin sulfate proteoglycan; (2) Mouse monoclonal antibody raised against 22–112 sequence of human SMA, which cross-reacts with rat (DAKO-Millipore); (3) Rabbit polyclonal antibody raised against native anti-Von Willebrand factor from human plasma and also recognizes the rat homolog; (4) Rabbit monoclonal antibody raised against a recombinant fragment 400–600 of mouse CD31 and cross reacts with the rat homolog; (5) Rabbit monoclonal antibody raised against the intracellular C-terminus of the human platelet-derived growth factor receptor beta (PDGFRβ) receptor, homologous to rat; (6) Rabbit polyclonal to Tie2 receptor, raised against synthetic peptide within human TIE2 amino acid 450–500 and cross reacts with rat, mouse, and human TIE2.

After washing, the cultures were incubated for 2 h at room temperature with the following secondary antibodies (1/200–1/500 dilution), solubilized in 2 ml of PBS with 0.1% Tween20, 1% FCS: (1) goat anti-rabbit IgG conjugated to fluorescein-isothiocyanate (FITC); (2) goat anti-mouse IgG conjugated to cyanine Cy5; (3) goat anti-mouse IgG conjugated to Cy3; (4) Donkey anti-rabbit IgG conjugated to Alexa Fluor 555.

Flasks were then washed and 4′, 6-diamidino-2-phenylindole (DAPI) was added to stain the nuclei. The cells were then examined under a fluorescence microscope Zeiss Axiovert 40 CFL or Zeiss AxioObserver A1 fitted with AxioCam HRc and AxioCam MRm cameras, respectively. For Alexa Fluor 555 staining analysis, images were further processed with self-written SynoQuant Ver. 10.69 software (for details see Henkel et al., 2019). The images were deblurred with a non-linear algorithm (“Level background”) that removed specifically homogenous stain corresponding to background staining, while enhancing structured any objects. The separation between background and structures was based on a local intensity standard deviation.

### Oxygen Glucose Deprivation Protocols

A glove box (Plas BY Labs, Lansing, MI, USA) was maintained with 0% O_2_, 5% CO_2_, and 5% H_2_ in N_2_ at 37°C (for the justification of gas composition see Al-Sarraf et al., 2018). All cell culture media and buffers were kept in the glove box for at least 24 h prior to OGD experiments. Cell cultures were incubated in glucose- and pyruvate-free DMEM (Invitrogen) that did not contain other supplements (OGD medium) for 2–24 h. For control experiments, cultures were incubated for 2–24 h in supplement-free DMEM that contained 5 mM glucose and 1.25 mM pyruvate (control medium) in 5% CO_2_ in air at 37°C.

In some cases, the following cytokines were added to the cell culture medium prior to the start of the experiments: (1) Rat recombinant EPO (rrEPO, R&D Systems, UK), 5 IU/mL, corresponding to ≈40 ng/ml; (2) Rat recombinant vascular endothelial growth factor (rrVEGF_A1−165_, Abcam, UK) 10 ng/ml; (3) Rat recombinant Angiopoietin 1 (rrAng1, My BioSource, UK) 40 ng/mL.

The rationale behind choosing these cytokines was that they have been shown to be the three main cytokines released from cells of the neurovascular unit during hypoxia (Augustin et al., 2009; Becerra-Calixto and Cardona-Gómez, 2017; Hu et al., 2017). Final concentrations of these three cytokines were selected based on the available data. The concentration of rrEPO was selected according to its determined ED_50_ value for the induction of erythroleukemic TF-1 cell line proliferation (Krejci et al., 2009) and on its concentration in the cerebrospinal fluid (Janik et al., 2010). The concentration of rrAng1 was selected according to the manufacturer's recommendation, Ang1 activation of Tie2 receptor (Bogdanovic et al., 2006) and on Ang1-induced proliferation of endothelial cells (Kanda et al., 2005). Concentration of rrVEGF_A1−165_ was chosen according to the manufacturer's recommendations, dose-dependent effects of VEGF_A1−165_ on BECs (Mayhan, 1999) and on its concentrations in the rat CSF (Jones et al., 2003). Also, observed effects of these cytokines on cell viability and apoptosis (Koblizek et al., 1998; Kwak et al., 1999; Papapetropoulos et al., 2000) were taken into consideration.

### Assessment of Cell Viability and Apoptosis

Following completion of anoxia or control protocols, cells were detached, stained with Annexin–V PE and 7-aminoactinomycin D (7-AAD) and analyzed using Epics XL flow cytometer (Beckman Coulter, USA). Based on the staining cells were considered as viable, apoptotic or dead (see Supplemental Figure 2). A marginal fraction of the cells in every sample was 7-AAD-positive/Annexin PE-negative; since this fraction was independent of experimental procedure it was excluded from the analysis.

### Assessment of Cell Contractility and Motility Speed

We measured the membrane dynamics and the overall contraction/relaxation of pericytes. The measurements were done on digitally segmented single cells or small connected cell groups (<3 cells). **Figure 3E** and Supplemental Figure 3 show representative examples of pericytes in contracted and relaxed states. Additionally, we quantified the overall motility speed upon various experimental interventions in order to determine if the cellular movement apparatus was affected by the treatments. The Supplemental Video 1 shows the typical morpho-dynamics of a small connected group of pericytes. Contraction and dynamics of large processes are quantified by the area/perimeter ratio (A/PR), while small membrane movements could be measured by the fractal dimension (D_f_) of the cellular perimeter (red border around cells in Supplemental Figure 3). The mobility speed was quantified by spatial intensity difference changes between successive image frames; this protocol was described in detail previously (Henkel et al., 2019).

Primary cultures in flasks were transferred to a temperature–controlled (37°C) incubation microscope (Cell Observer, Zeiss, Germany). Image sections for morpho-dynamic analysis were selected, if they contained preferentially single cells, covering not more than 50% of the area. Control images were recorded at a rate of 1 per 5 min for 15 h. The culture flasks were then transferred to the glove box and exposed to OGD for variable time periods (20 min−6 h). The flasks were then transferred back to the cell observer and the same cells that were imaged before OGD were monitored again. Thus, during this second imaging protocol, cells were exposed to control conditions. Image stacks of 11–22 cells from different flasks were obtained for each experimental group before and after OGD for statistical analysis.

### Image Acquisition, Processing, and Quantification

Details of all procedures have been described previously (Henkel et al., 2019). The following parameters were measured: single cell membrane mobility (SCMM), Area/Perimeter ratio (A/PR), which corresponds to cell surface–cell volume ratio and is well-suited to monitor cell process dynamics. Finally, the fractal dimension (D_f_) was calculated using the segmented cell perimeter as an object, which provided a quantitative approximation of the cellular plasma membrane complexity (Popescu et al., 2010).

Co-localization of immune-stained proteins (green and red channels) was measured with SynoQuant's build in “Co-localization module” that included a background subtraction algorithm, which was based on structure elements in the image. Essentially, the analysis employed the Pearson's correlation coefficient between the red and green channels (Dunn et al., 2011) to calculate coincidental appearances of proteins inside a cell and cell structures.

### Statistical Analysis

The value distribution was assessed by Shapiro-Wilk's test for normality (Shapiro and Wilk, 1965; Villaseñor and González-Estrada, 2009). Differences between experimental groups were pre-assessed by one-way or two-way ANOVA to determine the effects of OGD, cytokine presence, and viability of pericytes. The homogeneity of variances was assessed by Levene's test for equality of variances (Levene, 1960). Whenever the data was not normally distributed, the Kruskal-Wallis test was used instead of ANOVA.

For assessment of the contractility, statistical significance was calculated by paired *t*-tests between control and experimental groups, since the same cells were imaged before and after OGD. A *p* < 0.05 was considered statistically significant. Effect sizes between controls and experimental groups were calculated as Cohen's D (Cohen, 1988).

## Results

### Purity of Primary Cultures

Overall, rat brain pericytes in culture revealed heterogeneous phenotypes and were the largely dominant cell type, while VSMCs were only marginally present.

**Figure 1:**
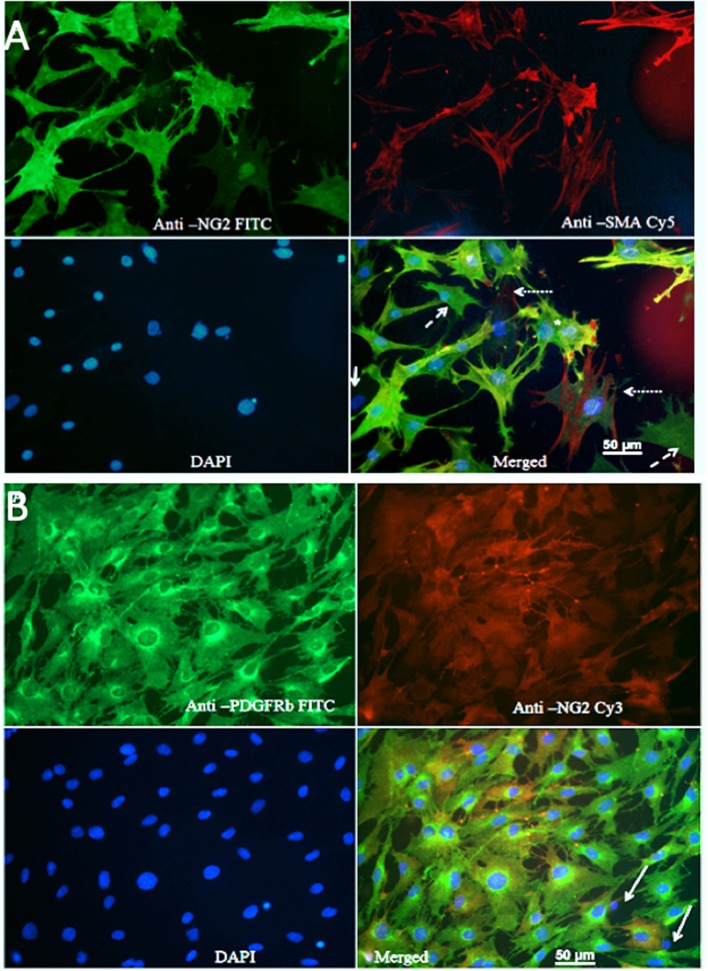
**(A)** Staining of primary cultures with anti-NG2 (FITC, green channel)/anti-SMA antibodies (Cy5, red channel). These images show polygonal, pericyte-like cells with processes. Anti-NG2 was mostly diffuse diffusely, while staining with anti SMA revealed fibrillary structures, which most likely corresponded to actin filaments. Cells that were poorly stained with both NG2 and SMA could be observed rarely (arrows); some cells were positive only for NG2 (dashed arrows), while some cells were mainly SMA positive (dotted arrow). **(B)** Immunostaining for PDGFRβ. A typical image showing staining of primary cultures with anti PDGFRβ (FITC, green)/anti-NG2 antibodies (Cy3, red).

Figure 1A shows that the majority of cells were NG2-positive (FITC), and many NG2-positive cells were also positive for SMA (Cy5). The Pearson's coefficient for cellular co-localization of both markers, after background correction, was ≈ 0.7, which corresponds to a colocalization of ≈ 48–50%. Cells with a typical pericyte-like morphology that were positive for NG2 and marginally positive for SMA could be observed in all cultures (dashed arrows). Cells that were positive for SMA and marginally positive for NG2, and thus could represent vascular smooth muscle cells (dotted arrows). Rarely, cells showed no immune-reactivity for either NG2 or SMA (solid arrows); these were possibly fibroblasts or macrophages. Since expression of SMA by pericytes largely depends on their localization, pericytes from capillaries that branch directly from arterioles and venules express more SMA than those in mid-capillary regions (Nehls and Drenckhahn, 1991). This may account for the absence or low abundance of SMA in pericytes from these areas. Contamination of primary cultures with BECs was marginal, which was confirmed by immunostaining with anti-Von Willebrand factor antibody and CD31 antibody (Supplemental Figure 4).

Since brain pericytes can be distinguished from VSMCs by PDGFRb as marker (Hutter-Schmid and Humpel, 2016), we stained primary cultures with anti-PDGFRb (FITC, green channel) and anti-NG2 (Cy3, red channel) antibodies (Figure 1B). The vast majority of cells were PDGFR-positive; the Pearson's coefficient for co-localization of both markers after background correction was ≈ 0.7, which corresponded to a co-localization of ≈ 48–50%. PDGFRb positive–NG2 negative cells were present in all cell cultures. A small fraction of the cells was NG2 positive but PDGFRb negative, presumably VSMCs. Isolated pyknotic nuclei were also observed (arrows).

### Viability of Pericytes During OGD and Effects of NVU-Derived Cytokines

**Figure 2:**
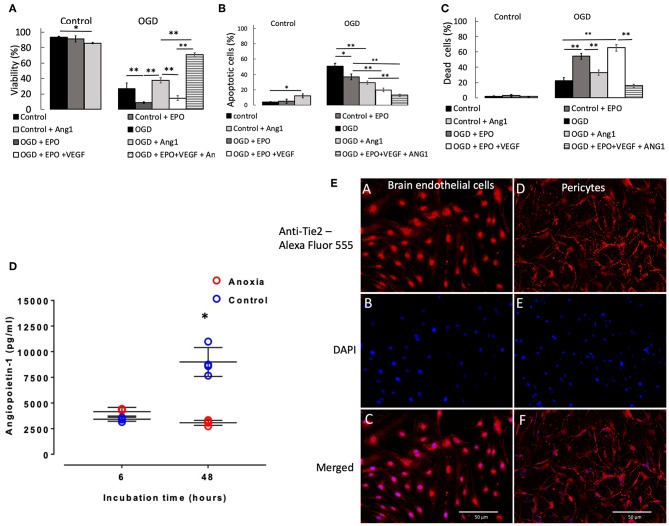
Percentage of pericytes that were viable **(A)**, apoptotic **(B)**, and dead **(C)**, respectively after 24 h control or OGD protocols. Data were presented as mean ± SD, *n* = 4–8. Symbols indicate: *p < 0.05, **p < 0.01. **(D)** Angiopoietin-1 secretion by pericytes in culture during anoxia and control conditions. Values from each sample are presented as separate points. In some cases, data overlap and hence may appear to be <4 samples. The mean of the four values and SD were calculated; the latter ones being presented with solid lines. In some cases, SDs were marginal, so they appear as a single line. Symbol [*] indicates that values in anoxic groups were lower (*p* < 0.05) vs. values during normoxia (control). **(E)** Staining of rat primary brain endothelial cells (left column of images, A–C) and rat primary pericytes (right column of images, D,E) for Tie2 receptor. (A,D) Anti-Tie2 receptor staining, labeled with Alexa Fluor 555; (B,E) DAPI stain; (C,F) overlay of anti-Tie2 receptor and DAPI staining.

Two and six hours OGD caused small but significant (*p* < 0.05) reduction in pericyte viability (Supplemental Figure 5A), which was accompanied by a small increase in the number of apoptotic (Supplemental Figure 5B) and dead cells (Supplemental Figure 5C). However, 24 h exposure to OGD caused a large reduction in viability, to 27% (*p* < 0.001 vs. control, Figure 2A), which was accompanied by a large and significant increase in the number of apoptotic cells (Figure 2B) and a moderate and significant increase in the number of dead cells (Figure 2C).

Presence of rrEPO in in the medium did not exert any effect on cell survival either during 2 and 6 h OGD or in control conditions during 2, 6, and 24 h (Figure 2A and Supplemental Figure 5A). However, it caused a large decrease in cell viability after 24 h OGD from 27% (OGD without rrEPO) to 8% (*p* < 0.01, Figure 2A). The number of apoptotic cells significantly decreased under these conditions to 35% (*p* < 0.05 vs. OGD without rrEPO, Figure 2B), but the number of dead cells increased significantly to 55% (*p* < 0.01 vs. OGD without rrEPO, Figure 2C). Interestingly, presence of rrAng1 in in the medium caused a marginal and significant reduction in cell viability in control conditions (*p* < 0.05 vs. control without any cytokine, Figure 2A), which was mainly due to a significant increase in the number of apoptotic cells (Figure 2B). This finding requires further investigation of the mechanism behind it, however, this was beyond the scope of the current study. Presence of rrAng1 in in the medium caused a marginal and insignificant increase in cell viability during 24 h OGD, when compared to OGD without any cytokine added (*p* = 0.247, Figure 2A). When rrVEGF_A1−165_ and rrEPO were added together to the cell culture medium, there was a decrease in viability of pericytes after 2 h OGD to 71% (*p* < 0.01 vs. OGD and vs. OGD + rrEPO, Supplemental Figure 5A) and an increase in cell viability after 6 h OGD to 95% (*p* < 0.05 vs. OGD and vs. OGD + rrEPO), while after 24 h OGD cell viability under these conditions was only 14%, which was significantly lower than after 24 h OGD without any cytokine added (*p* < 0.01, Figure 2A). This was mainly due to a large increase in the number of dead cells (Figure 2C). Addition of rrEPO, rrVEGF_A1−165_ and rrAng1 to the cell culture medium before exerted only small effects on cell viability after 2 and 6 h but were strongly protective to pericytes after 24 h OGD, with a cell viability exceeding 70% (*p* < 0.001 vs. OGD without cytokines, Figure 2A). This was due to a large reduction in the number of apoptotic cells (<13%, Figure 2B) and dead cells (<16%, Figure 2C). This observation suggests that a large number of pericytes could survive as long as 24 h without oxygen, glucose and pyruvate in the presence of these three NVU-derived cytokines. Since pericytes normally secrete Ang1 we speculated on why this secretion could not exert protective effects when primary pericytes were exposed to OGD with rrEPO and rrVEGF_A1−165_, i.e., why was the difference in the survival between these two groups (rrEPO and rrVEGF_A1−165_ vs. rrEPO, rrVEGF_A1−165_ and Ang1) so large. To clarify this, we used ELISA kit (Boster biological technology, USA) to measure concentrations of Ang1 in cell culture media collected from the conditioned pericytes. These results are presented in Figure 2D. In the presence of oxygen, pericytes secreted Ang1 in the medium and there was an almost linear increase in the concentration between 6 and 24 h in the control groups. However, when they were deprived of oxygen, there was no significant increase in Ang1 concentrations between 6 and 48 h (*p* > 0.05); the concentration after 48 h was significantly lower than in controls (*p* < 0.01).

After staining for Tie2 receptor, immunofluorescence showed punctate staining on the membrane in both brain endothelial cells, which were used as a control, and in primary pericytes (Figure 2E). There was some nuclear staining that was more prominent in endothelial than in pericyte cells. Pericyte processes were more intense stained than cell somata, while endothelial cells showed a more homogeneous distribution and smaller puncta.

### Effects of OGD Protocols on Pericytes' Morpho-Dynamics

**Figure 3:**
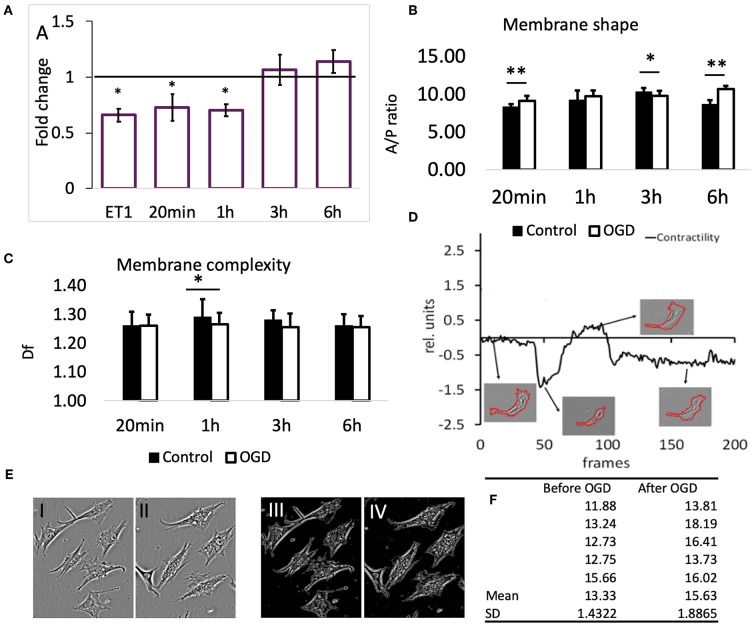
**(A)** Effects of OGD protocols on cell membrane mobility. The bars represent the fold-change compared to controls of motility speed after exposure to OGD for the indicated time periods. ET1 serves as a positive control for motility speed reduction. Data are presented as mean ± SD from the following number of cells: 22 (ET-1), 11 (20 min), 16 (1 h), 22 (3 h), and 16 (6 h). **(B)** Membrane shape of pericytes before and after OGD quantified by area/perimeter ratio (A/PR) at indicated OGD exposure times. The values were obtained from the following number of single cells: 25 (20 min), 22 (1 h), 18 (3 h), 20 (6 h). **(C)** Membrane complexity (membrane roughness) of pericytes, represented by D_f_, before and after OGD protocols. Asterisk indicates significance level to corresponding controls (set to 1) *p < 0.05, **p < 0.01. **(D)** Example of a contractility time series derived from a contraction-expansion cycle of a single pericyte. The insets show the outlines of pericyte plasma membranes at the indicated time points in the contractility trace. **(E)** An example of a partial retractions of cell processes after OGD. I and III show a typical visual field under phase contrast microscope with 6 pericytes before, while II and IV show the remaining 5 pericytes after 6 h OGD. Images III and IV represent structure enhanced images I and II, respectively. **(F)** Using protocol that has been described already (Henkel et al., 2019), the A/P ratio for every cell in images shown in E has been calculated and then the mean and SD estimated. These data reveal that A/P ratio for each of the five cells in this particular visual field has increased, i.e., cells have partially retracted processes becoming more “round.” Paired *t*-test revealed statistically significant difference (*p* < 0.05).

To confirm that the reduction in SCMM actually represented contraction of the contractile filaments in pericytes, 22 cells were imaged before and after application of the vasoactive drug endothelin-1 (ET-1, 50 nM) (Rubanyi and Polokoff, 1994). The result is presented in Figure 3A, first bar; 19 out of 22 cells exhibited a reduced SCMM (*p* < 0.01, *n* = 22).

Exposure of pericytes to 20 min (*n* = 11) and 1 h (*n* = 16) OGD produced a similar effect as the presence of ET-1, which was a pronounced reduction of pericytes' SCMM when compared to the corresponding pre-OGD control period (Figure 3A, *p* < 0.05 for both time points). The effect sizes (Cohen's D) after 20 min and 1 h OGD, were 0.65 and 0.9, respectively, corresponding to medium and large effects. After 3 and 6 h OGD, the overall motility speed of their processes did not change significantly, when compared to the pre-OGD period (*n* = 22, *p* = 0.02 and *n* = 16, *p* = 0.46, respectively). In both cases the effect sizes were small (Cohen's D < 0.4).

The ratio between cell area and cell perimeter (A/PR) was used to check if the protrusions and process formation was affected by OGD. Initially, the A/PR increased after 20 min OGD (Figure 3B, *n* = 25, *p* < 0.01), which indicated that the cell became rounder and that the processes grew shorter. Later on, there were either no changes detectable (1 h, *n* = 22), or a slight decrease (3 h, *n* = 18, *p* < 0.05) compared to controls. The exposure to 6 h OGD caused a significant increase in A/PR (*n* = 20, *p* < 0.01), pointing to the retraction of processes and protrusions. In summary, the pericytes became rounder after 20 min and 6 h OGD.

Generally, we could not detect differences in pericytes' membrane complexity estimated by fractal dimension D_f_ (Figure 3C) between images taken before and after OGD, except when cells were exposed to 1 h OGD, when D_f_ was significantly decreased (*p* < 0.05 compared to controls, *n* = 54). In order to verify that this reduction was associated with contraction of cellular processes, we measured D_f_ before and after addition of ET-1 (50 nM). Despite heterogenous behavior of pericytes, ET-1 caused a significant reduction in D_f_ from (mean ± SD) 1.28 ± 0.04 before OGD to 1.25 ± 0.05 after OGD (*n* = 65, *p* < 0.01).

In most pericytes the plasma membrane motility was very dynamic, but on a slow time scale. Figure 3D (modified from Henkel et al., 2019) shows a representative A/PR trace with transient contractions and relaxations, occurring over the course of ≈15 h. At the end, most pericytes remained rounder than at the start of the experiment. The 4 insets show the segmented cell plasma membrane, acquired at corresponding times (arrows) of the experiment.

## Discussion

### Pericytes Were Resistant to OGD

The data from this study suggest that pericytes in primary culture represented a heterogenous population of cells; they could survive OGD for relatively long periods of time, if rrEPO, rrVEGF, and rrAng1 were added in the cell culture medium. The only source of glucose during OGD could be from glycogen. In the presence of NVU-derived cytokines, astrocytes store substantial amounts of glycogen and metabolize it by glycolysis (Rischke et al., 1992; Thoren et al., 2006). Pericytes have been shown to store glycogen *in vivo* (Cataldo and Broadwell, 1986); our findings suggest that the available glucose quantity is apparently sufficient to supply ATP in the absence of oxygen.

### NVU-Derived Cytokines Exerted Differential Effects on Pericytes During OGD

EPO exerted detrimental effects on pericytes after 24 h OGD, which could be either due to a reduced ability of these cells to generate sufficient amounts of ATP through glycolysis, or to an increase in energy expenditure. These effects were similar to those observed on rat astrocytes and rat BECs during anoxia (Al-Sarraf et al., 2018) and contradict the classical concept that EPO signaling is neuroprotective during hypoxia (Mallet and Ryou, 2017). Astrocytes are the main source of endogenous EPO in the hypoxic brain (Chavez et al., 2006). Clinical trials have revealed either no effects or damaging effects of exogenous EPO in stroke patients (Souvenir et al., 2015). Cerebral glucose metabolic rate was significantly increased in constitutively EPO-overexpressing transgenic mice (Frietsch et al., 2007). This suggests that EPO enhances energy demands in brain cells. EPO is also one of the cytokines that could affect cellular metabolism through a 5′ AMP-activated protein kinase (AMPK–EC 2.7.11.31)—dependent pathway (Wang et al., 2013). AMPK activation in pericytes enhances glucose uptake by increasing the expression of GLUT-1 and GLUT-4 and ATP production through glucose utilization (Carlsson et al., 2018). This could enhance glucose utilization beyond the minimum required and, therefore, could reduce ability of pericytes to survive OGD for a longer period of time. Activation of AMPK was detrimental to neurons during ischemia-reperfusion, while AMPK inhibition was neuroprotective (Ronnett et al., 2009). VEGF can also activate AMPK (Baufeld et al., 2017; Zibrova et al., 2017) and pericytes express VEGF receptor 1 (Eilken et al., 2017). Thus, it is likely that the detrimental effects of simultaneous application of VEGF and EPO on pericytes during OGD could be due to enhanced AMPK activation, which in turn enhances glycolysis, and depletes available energy substrate (i.e., glycogen) more rapidly, while increasing lactate production. An increase in lactate inhibits Na^+^/Ca^2+^ exchangers (Yamanishi et al., 2006), which triggers Ca^++^ overload and apoptosis.

The presence of rrAng1 in the medium, though it significantly reduced the number of dead cells (Figure 2C), exerted only marginal and insignificant increase in viability of pericytes during 24 h OGD. On the other hand, presence of rrEPO, rrVEGF_A1−165_, and rrAng1 in the cell culture medium exerted protective effects on pericytes during 24 h OGD. It is not clear why this protective effect was so different, when compared to the effects observed in the presence of rrEPO alone, rrAng1 alone or rrEPO and rrVEGF. One of the explanations could be that pericytes secrete Ang1 during hypoxia, but in the absence of oxygen pericytes cannot continue to produce Ang1 for a longer period of time (Figure 2D); thus, the protective effect appeared only when rrAng1 was added to the medium. Ang1 exerts its effects by binding to the Tie2 receptor tyrosine kinase (Augustin et al., 2009; Reiss et al., 2015), which is primarily expressed in endothelial cells (Sato et al., 1995), but this study (Figure 2E) confirmed previous findings that it was also expressed in pericytes (Teichert et al., 2017). The only common intracellular mechanism that is activated by EpoR, VEGFR, and Tie2 is the phosphatidylinositol 3-kinase pathway (PI3K, EC 2.7.1.137). Subsequent stimulation of 3-phosphoinositide-dependent kinase 1, activates RAC-alpha serine/threonine-protein kinase (AKT1). The resulting phosphorylation suppresses apoptosis by inhibiting the Bcl-2-associated death promoter through the p53 protein. It has been shown that a well-known β-adrenergic receptor agonist that exerts protective effects on retinal pericytes in diabetes patients is mediated *via* Akt activation (Yun et al., 2018). Thus, we can assume that simultaneous activation of the PI3K pathway by the three cytokines, mentions above, reduces apoptosis of pericytes during OGD, similar to earlier findings that have shown PI3K/Akt dependent apoptosis reduction in retinal pericytes (Haribalaganesh et al., 2009).

### Do Pericytes Contract During OGD?

In this study, we have video-imaged pericytes, exposed them to OGD of various durations and then video-imaged the same cells again in normal conditions, which partially mimicked conditions that occur during the reperfusion *in vivo*.

Addition of ET-1 to primary cultures caused changes in SCMM and D_f_ that corresponded to cell contractions, which confirms that the vast majority of pericytes in primary cultures, regardless of their heterogeneity, possess the necessary cellular apparatus to contract. OGD protocols that lasted for 20 min and for 1 h caused changes in pericytes' morphology similar to changes caused by addition of ET-1. However, they did not persist after 3 and 6 h, though the latter data was not conclusive, since changes in D_f_ were consistent with contraction of processes. We observed that 20 min OGD protocol caused a significant reduction in cell mobility. This was even more pronounced after 1 h OGD protocol. These effects disappeared after 3 and 6 h OGD, which suggests that the mobility of the cells was transiently impaired by OGD. The A/PR increased after 20 min and 6 h OGD, indicating rounder cells with fewer processes. Interestingly, since 1 and 3 h long exposure to OGD had no apparent effects on process formation, it appears likely that two independent molecular mechanisms may control the membrane shape. Overall, it appears that brain pericytes do contract after 20 min and possibly 1 h OGD protocols, but not after 3 h and also most likely after 6 h OGD protocols.

## Limitations and Conclusions

Our results revealed that OGD transiently affected cell mobility and cell shape, which was followed by cell apoptosis in cultured pericytes. Angiopoietin-1 may have a potentiality for preventing from the OGD-induced apoptosis. However, further analysis is required to clarify the relationship between cell contraction and cell apoptosis during OGD. The main limitation of this study was that it was an *in vitro* study, hence these findings should be applied to *in vivo* conditions with caution. Also, SCMM has not been determined in the presence of Ang1, VEGF, and/or EPO. If the time pattern of pericytes' survival and contraction *in vivo* is the same as revealed in our *in vitro* study, for which we have no evidence so far, then it is unlikely that rapid death and contraction of pericytes are the cause of no-reflow in the brain.

## Data Availability Statement

The datasets generated for this study are available on request to the corresponding author.

## Ethics Statement

The details of the experimental protocol were approved by the Animal Ethics Committee, HSC.

## Author Contributions

MH performed the study of pericytes' viability and apoptosis during OGD and analyzed these data. LA-A performed the study of pericytes' motility and contractility after OGD and analyzed these data. AH developed the software to analyse motility and contractility data, supervised data analysis, and partially wrote the manuscript together with ZR. ZS participated in all cell culture work and performed NG2/SMA/PDGFRb staining. SM performed staining for Tie2 receptor in pericytes and BECs. ZR designed and supervised the study, provided funding, supervised data analysis, directed other contributors, and drafted the manuscript together with AH.

### Conflict of Interest

The authors declare that the research was conducted in the absence of any commercial or financial relationships that could be construed as a potential conflict of interest.

## Footnotes

^1^Available at https://olaw.nih.gov/sites/default/files/GuideBook.pdf

^2^Available at https://www.nc3rs.org.uk/sites/default/files/documents/Guidelines/Responsibility%20in%20the%20use%20of%20animals%20in%20bioscience%20research-%20expectations%20of%20the%20major%20research%20councils%20and%20charitable%20funding%20bodies%20April%202017.pdf

## Supplementary Material

Supplementary Video 1 Time lapse video of contracting and expanding pericyte in primary culture.
