# Supplementary material for: Viability and Contractility of Rat Brain Pericytes in Conditions That Mimic Stroke; an in vitro Study
Source: Front Neurosci. 2019 Dec 5;13:1306. doi: 10.3389/fnins.2019.01306 (PMC6906154; doi:10.3389/fnins.2019.01306)
Supplement: Supplementary file 1 [file Table_1.DOCX]

Supplemental figure 1


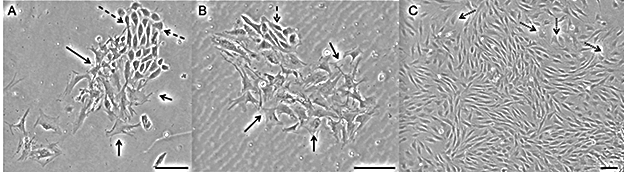


Phase contrast microscopy of cells in the primary culture at day 3 (**A**), 5 (**B**) and day 3-4 after the passage (**C**). Bars indicate 50 µm.

The digest of the brain microvessels was seeded on rat collagen 1 – coated plastic in the medium that did not contain puromycin, an amino-nucleoside antibiotic and a P-glycoprotein substrate that was shown to be toxic in high doses for non-endothelial cells in culture (Perrière et al, 2005). Thus, initially, both BECs (as cell islands dashed arrows) and pericytes (sloid arrows) grew in the culture (**A**). After 5-6 days in culture, the endothelial cell islands growth was reduced and a large number of pericytes could be observed in culture (**B** -arrows).

Cells were then passaged to uncoated plastic in order to eliminate BECs from the culture. The rationale behind this approach was that pericytes produced sufficient amount of the basal lamina components, to attach onto uncoated plastic and survive, while BECs were not able to do so (Abbott et al., 2012). Some cells were large, with >50 µm in diameter (dotted arrows) with multiple processes spreading in all direction, while majority of cells were smaller, but still revealed many processes and were polygonal in shape.

Supplemental figure 2


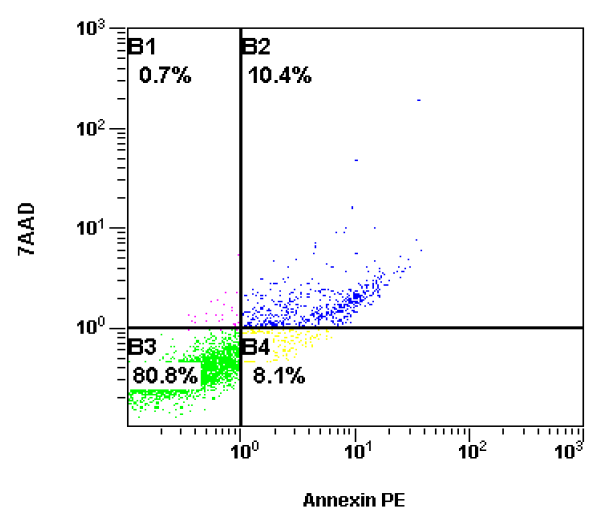


A typical flow-cytometry correlation diagram. X-axis and Y-axes indicate quantities of annexin PE and 7-Aminoactinomycin D (7-AAD) fluorescence, respectively. Viable cells were negative for both dyes and located in B3 quadrant (80.8%); apoptotic cells were positive for annexin PE and negative for 7-AAD and located in B4 quadrant (8.1%). Dead cells were positive for both dyes and located in B2 quadrant (10.4%). A marginal fraction (<1%) of the cells in every sample was 7-AAD-positive / Annexin PE-negative and located in the upper left quadrant.

Supplemental figure 3


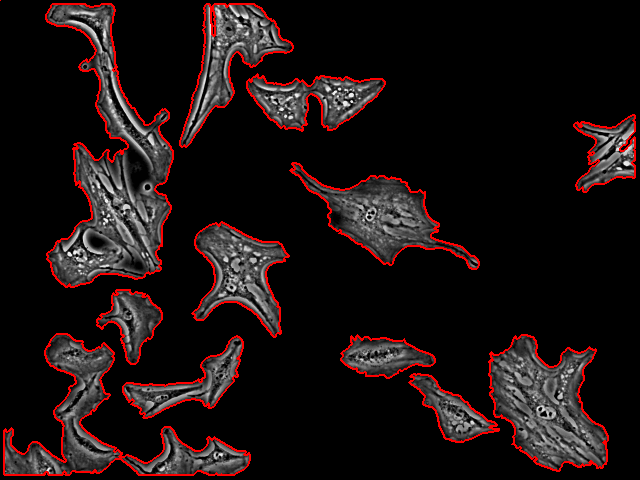

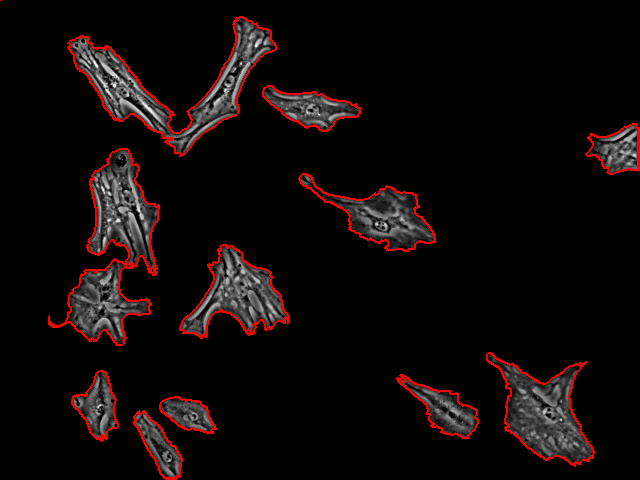


Contracted pericytes

Relaxed pericytes

25 μm

25 μm

#### Cell segmentation

Representative images of contracted (upper panel) and relaxed pericytes (lower panel). The plasma membrane of the cell is segmented by a red line, using SynoQuant imaging software (SynoSoft, Kuwait).


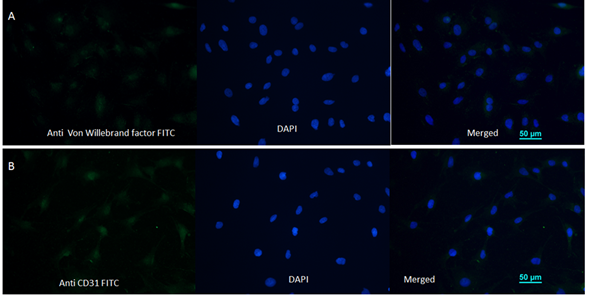
Supplemental Figure 4

1


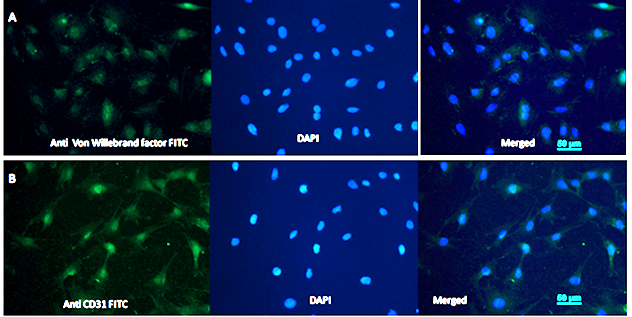


2


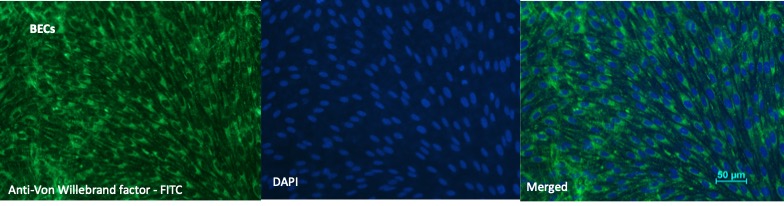


3

**C**

Panel 1: Staining of primary cultures with antibodies against markers for endothelial cells, anti Von-Willebrand factor (**A**) and anti CD-31 (**B**) In both cases, a weak staining was observed, with a vague perinuclear staining and sometimes diffuse cellular staining. Panel 2is digital enhanced copy of the one above. Panel 3 shows staining of BECs in primary culture with vW antibodies - FITC (for the method see Redzic et al., 2015), which showed an intense staining.

Supplemental figure 5


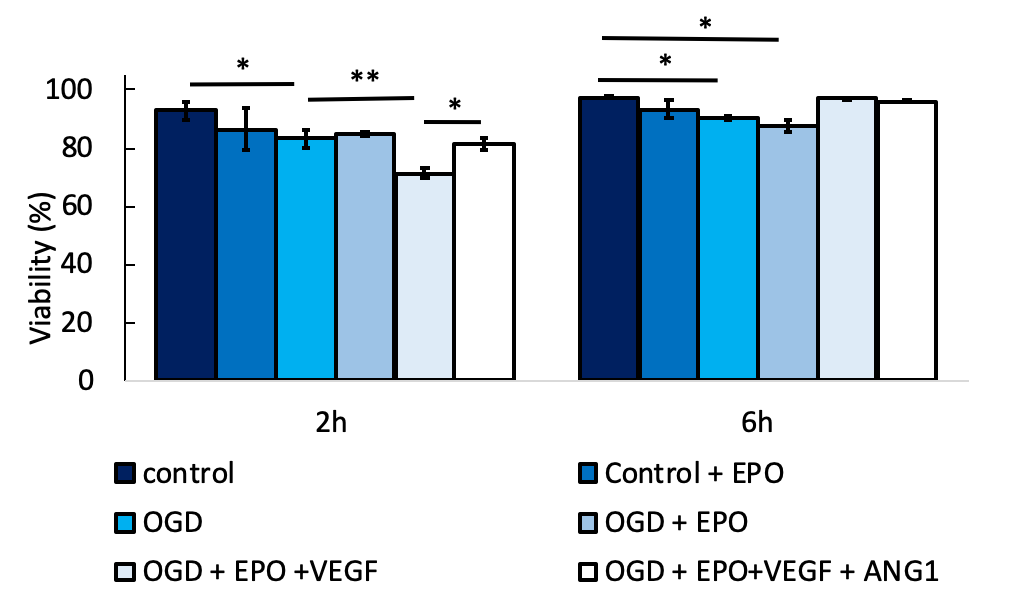
A


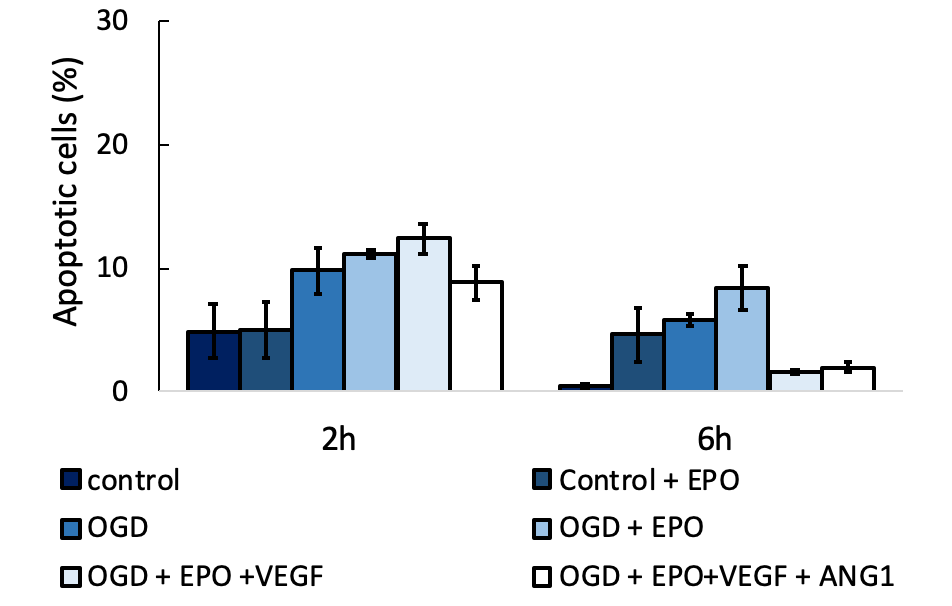
B


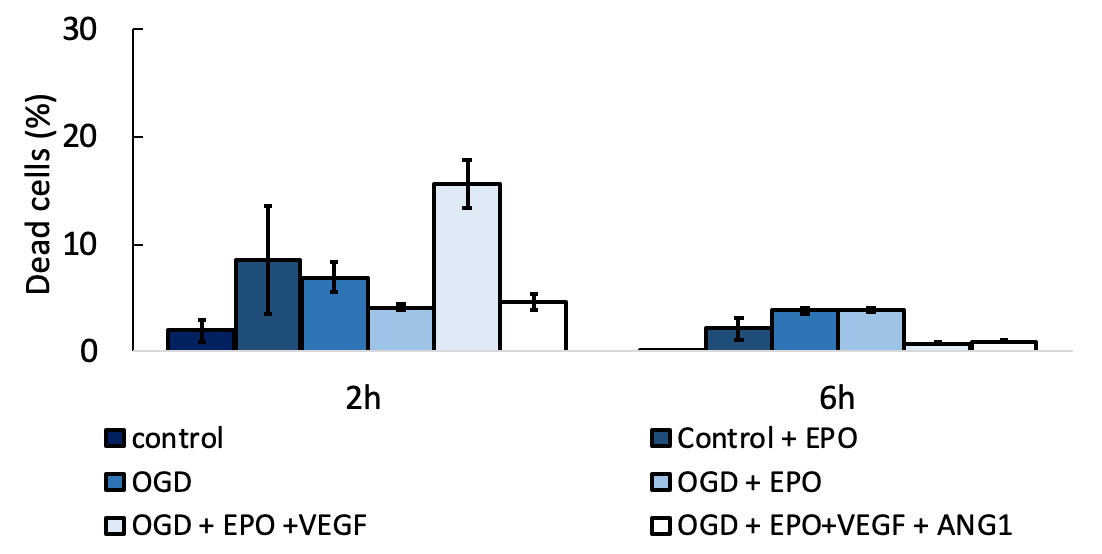
C

Number of primary pericytes (%) that were viable (A), apoptotic (C) and dead (D), respectively after 2h and 6h control or OGD protocols.
